# Public perception of air pollution sources across Europe

**DOI:** 10.1007/s13280-020-01450-5

**Published:** 2020-12-31

**Authors:** Michela Maione, Elisabetta Mocca, Kristina Eisfeld, Yuri Kazepov, Sandro Fuzzi

**Affiliations:** 1 Department of Pure and Applied Sciences, University of Urbino, Piazza Rinascimento 6, 61029 Urbino, PU Italy; 2 Institute of Atmospheric and Climate Sciences, National Research Council, Via Piero Gobetti, 101, 40129 Bologna, Italy; 3 Department of Sociology, University of Vienna, Rooseveltplatz 2, 1090 Vienna, Austria

**Keywords:** Air quality, Behavioural changes, Environmental policies, Social acceptability

## Abstract

Air pollution is one of the primary concerns of our society for its effect on human health and the environment. Among the policy measures that can be put in place to limit air pollutant emissions, end-of-pipe technologies and/or regulatory instruments may be implemented through legislative acts. Also, equally important are behavioural measures, requiring citizens’ active involvement. The success of any measure to limit pollutant emissions requires the acceptance by the citizens that, in turn, implies a correct perception of the main pollutant emission drivers. We present here the comparison between the public perception of air pollution sources and the real-world situation through a survey carried out in seven European countries and involving 16 101 respondents. Our study shows a dramatic underestimation of the contribution of the agri-food sector to air pollution. This result is common to all respondents in the seven countries examined and only to a small extent depends on gender, age and socio-economic status of the respondents.

## Introduction

Non-communicable diseases (NCDs) are the leading cause of death in today’s world. After tobacco smoking, air pollution exposure is the second biggest environmental risk of NCDs globally (World Health Organisation 2019). This is because over 91% of people worldwide breathe air with pollution levels above air quality guidelines set by the World Health Organisation (WHO). The WHO estimated that ambient air pollution alone was responsible for approximately 3.5 million premature deaths worldwide in 2016 (Health Effects Institute 2018), with around 480 000 in the European Union countries alone (European Environment Agency 2018a).

Within the European Union legislative framework, atmospheric pollutant emissions are targeted by the revised National Emission Ceiling Directive (NECD) (European Directive 2016/2284), entered into force on 31 December 2016. This directive sets the 2020 and 2030 emission ceilings for five main air pollutants: sulphur dioxide (SO_2_), nitrogen oxides (NO_X_), non-methane volatile organic compounds (NMVOCs), ammonia (NH_3_) and fine particulate matter (PM_2.5_). The directive transposes the reduction commitments for 2020 agreed by the EU and its Member States under the revised Gothenburg Protocol adopted in 2012 by the United Nations Economic Commission for Europe (UNECE) Convention on Long-Range Transboundary Air Pollution (CLRTAP). The reduction commitments agreed for 2030 are designed to improve air quality in line with the WHO guidelines, thus reducing the health impacts of air pollution by half compared with the base year 2005 (European Directive 2016/2284).

Among the measures that can be put in place to limit pollutant emissions, some are end-of-pipe technologies (e.g. catalytic converters, filtering systems, etc.), or regulatory and financial instruments (e.g. low-emission zones, subsidies, etc.), which may be implemented through legislative acts. Also, equally important are the behavioural changes, which require citizens’ active involvement (commuting habits, energy choices, waste disposal, dietary habits, etc.). The success of any measure, both legislative and behavioural, to limit pollutant emissions requires the acceptance by the citizens, what is commonly defined as social acceptability. Borrowing Fournis and Fortin’s (2017) definition, we define social acceptability as:

the process of collective assessment of a given project […], integrating a plurality of actors (stakeholders) and spatial scales (from global to local), as well as involving the specific trajectory (past, present and future) of a political group or policy (community/society).

Therefore, social acceptability primarily requires that individuals are able to grasp the complexity and usefulness of new technologies, legal interventions or behavioural changes. To enable people to properly evaluate all this, knowledge and information are a crucial asset, and individuals should have access to the necessary knowledge on the sources of air pollutants and the cost–benefit of any proposed innovation.

Despite its relevance, there is little large-scale quantitative research delving deep into the citizen’s perception of the sources of air pollutants. Some published studies have identified socio-demographic factors affecting the citizens’ perceptions of air quality, and it has been found that younger or older people, women, urban dwellers, people with higher levels of education, people with health problems (such as respiratory symptoms) are more likely to be aware of the implications of air quality issues (Elliott et al. 1999; Howvel et al. 2003; Oltra and Sala 2014; Yu 2014; Liao et al. 2015; Guo et al. 2016; Schmitz et al. 2018). However, the focus of the above contributions is almost exclusively on the awareness or level of concern about air pollution (Howvel et al. 2003; Saksena 2011), while far too little attention has been paid to the knowledge about the specific contribution of the various sources of pollutants to the degradation of air quality.

Previous research has also shown that people tend to have little knowledge about causes, evolution and sources of air pollutants. For instance, Smallbone (2012) found out that only 51% of respondents in a survey were able to name one or more air pollutants and that the most commonly known pollutants were carbon monoxide and carbon dioxide (the latter, by the way, being primarily related to climate change). Moreover, 54% of Europeans do not think they are informed enough about air quality in their own country (European Commission 2019).

Drawing on survey data from the EC-funded project SEFIRA (Socio-Economic implications For Individual Responses to Air pollution policies in EU + 27), aimed at examining individual preferences for environmental and air quality policies, this paper analyses the citizen’s perception of the major sources of air pollution. The survey results are then compared with real-world data and, to the best of our knowledge, this paper represents the first study addressing citizens’ perception on pollution sources in comparison with data derived from direct observations and scientific analysis.

## Materials and Methods

The SEFIRA survey was designed with the scope of analysing individual preferences for air quality policy drivers using a Discrete Choice Model (DCM) (Valeri et al. 2016). To support the analysis of the choice preference exercise, we included in the survey questions on the environmental perception that are the focus of this contribution. The survey was administered during summer 2015 and contains answers from 16 101 European citizens from seven European countries (Austria, Belgium, Germany, Italy, Poland, Sweden and the United Kingdom), using a Computer-Assisted Web Interviewing (CAWI) technique. The selection of European countries is based on the welfare typology of Esping-Andersen (1990) which distinguishes three welfare regimes: *social*-*democratic*, *conservative* and *liberal*. In order to consider more recent debates, we integrated this codification with both a *residual* (Andreotti et al. 2001) and a *post*-*socialist* welfare regimes (Fenger 2007). Underlying this choice is the assumption that each welfare regime produces different policy approaches to environmental issues. The selected seven countries thus represent different socio-economic and political patterns of the European society as well as differently polluted environments. Given the research objectives, a preliminary selection of a target population who both use cars/motorcycles for their urban movements and consume meat (beef, pork, lamb, horse) and/or milk and dairy products more than 4 days per month was made. The sample was stratified according to socio-demographic and territorial indicators and is representative for gender, age and level of urbanisation. The latter draws from the Eurostat’s ‘urban–rural typology’, which groups NUTS3 regions in three categories: ‘predominantly rural’, ‘intermediate’ or ‘predominantly urban’ regions.^1^

For the purpose of this paper, we focus only on people’s perceptions of air pollution sources. In the questionnaire, respondents could choose two main sources/sectors considered as primarily responsible for air pollution out of a list of six options (agriculture, industry, transportation, domestic heating, domestic waste and others^2^). The information used in this analysis includes age, gender, education level (low, middle, high) and place of residence (urban, rural and intermediate). Descriptive statistics that summarize the characteristics of the participants are reported in Table 1. Chi-square (*χ*^2^) test of independence was used to evaluate if participants’ age, gender, education level and place of residence are associated with the participants’ opinion on the most important sources of air pollution. A *p* value lower than 0.05 was considered to be statistically significant. In order to assess the substantive strength of the associations between people’s perceptions of air pollution sources and the respondent profiles, the effect size (*w*) is reported and refers to small (*d* = 0.1), medium (*d* = 0.3), and large (*d* = 0.5) benchmarks, as suggested by Cohen (1988) for contingency tables. We used the statistical software STATA 15 for the analyses of the data.

**Table 1:** Respondents’ descriptive statistics

	Austria	Belgium	Germany	Italy	Poland	Sweden	United Kingdom	Total
**Gender %**								
Male	48.43	48.83	49.35	47.89	46.22	49.52	50.57	48.69
Female	51.57	51.17	50.65	52.11	53.78	50.48	49.43	51.31
**Educational level %**								
Low	12.43	13.87	39.70	10.86	1.35	49.74	22.52	21.50
Middle	63.74	35.09	32.04	56.02	46.09	33.48	37.57	43.43
High	23.83	51.04	28.26	33.12	52.57	16.78	39.91	35.07
**Age %**								
18–34	33.48	28.57	23.96	27.68	35.52	25.91	24.65	28.54
35–54	40.65	37.65	35.61	44.11	39.43	33.52	37.30	38.33
55–65+	25.87	33.78	40.43	28.21	25.04	40.57	38.04	33.13
**Place of residence %**								
Primarily urban	34.65	68.35	44.00	35.81	25.57	20.26	71.74	42.80
Intermediate	26.91	23.13	40.39	43.94	32.48	56.96	25.22	35.59
Primarily rural	38.43	8.52	15.61	20.25	41.96	22.78	3.04	21.61
**Total (*N*)**	2300	2300	2300	2301	2300	2300	2300	16 101

Respondents are equally distributed between females and males and are mainly concentrated in the 35–54 age classes in all countries (Table 1). The oldest age group (55–> 65) can be found in Germany (40%) and Sweden (41%), while the youngest age group (18–34) is found in Poland (36%) and Austria (33%). With reference to the education level, a majority of respondents in Austria (64%) and Italy (56%) are concentrated in the middle education group (upper secondary school), while for Sweden they are in the lower education group, which includes primary school and lower secondary schooling (50%). In Germany, respondents are more distributed across all the education levels. Belgium and Poland report the highest number of respondents with higher level of education (> 50%). Polish respondents have the lowest frequency of low educational level (1%). A majority of Austrian (38%) and Polish (42%) respondents are living in a rural region, whereas the majority of respondents from Belgium (68%) reported to live in a city. Swedish (57%) and Italian (44%) respondents predominantly live in intermediate regions.

## Results and discussion

### The perception survey

**Fig. 1:**
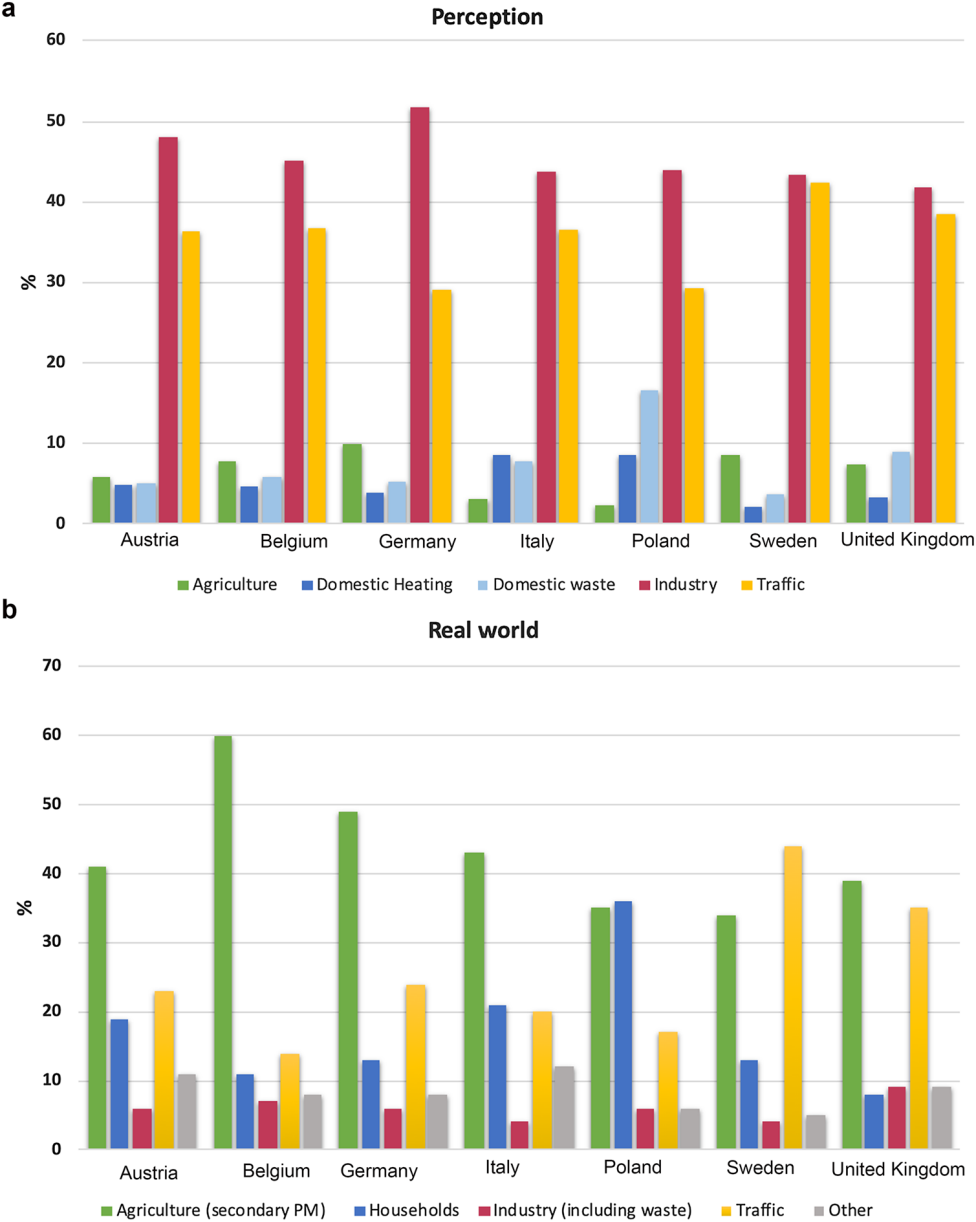
**a** Respondents’ perception of the most important air pollution sources in seven European countries. Source: SEFIRA survey. **b** Real-world emission sectors responsible, in percent, for urban PM_2.5_ atmospheric levels in seven EU countries. Data source: Kiesewetter and Amann (2014)

As far as the public perception of the main drivers of air pollution is concerned, the respondents could choose between five main sectors (agriculture, domestic heating, domestic waste, industry, traffic) as those primarily responsible for air pollutant emissions in their country. Each respondent was allowed to choose two sectors (multiple-answer categorization). Figure 1a shows the most frequently chosen air pollution sources for all seven countries. The data refer to percentages with respect to the overall sum of responses (26 790 answers in total).

Table 2 provides the segmented data according to country, gender, age, educational level and place of residence. The table shows that neither age, education, gender nor place of residence exhibits large differences on the emission sectors indicated by the respondents. Although *χ*^2^ results are significantly different from zero, the small effect sizes (*w*) indicate negligible differences among the different groups.

**Table 2:** Perceived atmospheric pollutant emission sectors in seven EU countries, segmented data (%). Source: SEFIRA survey. Number of respondents, *N *= 16 101

	Agriculture	Domestic heating	Domestic waste	Industry	Traffic
**Country**					
Austria	5.88	4.82	5.04	47.98	36.27
Belgium	7.83	4.61	5.87	45.04	36.66
Germany	9.92	3.94	5.31	51.73	29.10
Italy	3.19	8.59	7.74	43.84	36.54
Poland	2.35	8.50	16.67	43.94	29.29
Sweden	8.52	2.07	3.73	43.32	42.36
UK	7.45	3.30	9.00	41.76	38.49
*χ*^2^	269.9***	381.10***	735.28***	381.00***	305.00***
*w*	0.13	0.15	0.21	0.15	0.14
**Age**					
18–34	6.35	3.93	9.91	44.13	35.67
35–54	5.86	5.45	7.61	47.04	34.04
55–65+	6.9	6.12	5.8	44.29	36.88
*χ*^2^	8.67**	35.18***	123.18***	41.37***	31.44***
*w*	0.02	0.05	0.09	0.05	0.04
**Education**					
Low	6.36	2.91	6.49	47.84	36.40
Middle	5.63	5.17	8.25	45.69	35.26
High	7.21	6.63	7.71	43.33	35.11
*χ*^2^	25.02***	116.6***	26.56***	24.34***	2.20*
*w*	0.04	0.09	0.04	0.04	0.01
**Gender**					
Female	5.05	4.26	9.33	45.46	35.90
Male	7.73	6.26	5.94	45.11	34.95
*χ*^2^	76.85***	50.82***	124.95***	8.92**	12.15***
*w*	0.07	0.05	0.09	0.05	0.02
**Place of residence**					
Rural	5.22	5.49	8.99	45.60	34.70
Intermediate	6.19	5.06	7.21	45.30	36.23
Urban	7.01	5.29	7.36	45.11	35.23
*χ*^2^	17.71***	2.21*	25.14***	13.26**	7.33*
*w*	0.03	0.01	0.04	0.03	0.02

*p < .05; **p < .01;  ***p < .001; Cohen’s *w* < .1 small effect size, *w* < .3 medium effect size, *w* < .5 large effect size

As Fig. 1a and Table 2 show, industry and traffic are perceived as the most polluting sectors in all countries, while agriculture and households’ activities are seen as the least polluting ones. The highest differences among the countries are between Poland (2.35%) and Germany (9.92%) for agriculture, between UK (41.76%) and Germany (51.73%) for industry and between Germany and Poland (29.10 and 29.29%, respectively) and Sweden (42.36%) for traffic. The survey results (Table 1) also suggest that the higher the educational level of the respondents, the less industry is perceived as an important source of air pollutants.

### The real-world data

A rather different picture is obtained when considering real-world source sectors for PM_2.5_, here assumed to be a proxy for atmospheric pollution (Fig. 1b). The use of PM_2.5_ as a proxy is justified by the fact that most of the premature deaths in the EU-28 caused by air pollution are attributable to PM_2.5_, compared to other air pollutants (i.e. NO_2_ or O_3_) (see, e.g. Burnett et al. 2018). Indeed, this finding was also reiterated by a study recently published by the European Environment Agency (2019a). Real-world figures are based on data from the *Thematic Strategy on Air Pollution* (TSAP) Report (Kiesewetter and Amann 2014). This report quantifies the sources of urban PM_2.5_ levels in EU Member States, using the station-based modelling approach incorporated into the GAINS (*Greenhouse Gas—Air Pollution Interactions and Synergies*) modelling system (Kiesewetter et al. 2013). In this approach, PM_2.5_ is disaggregated into chemical, sectoral and spatial categories. Natural, transboundary transport, national, urban and local traffic emissions are considered.

The source sectors of the TSAP Report are not exactly the same as in the SEFIRA survey. Primary PM sources considered here are industry and traffic, while secondary PM sources are computed as the sum of the SO_2_ and NO_*X*_ industry contribution and NO_X_ traffic emissions, combined with ammonia emissions. The so-called secondary atmospheric aerosol, i.e. atmospheric particles that are not emitted as such (primary aerosol) but are formed in the atmosphere by reactions of gaseous precursors, constitutes a relevant fraction of PM_2.5_ concentration in most parts of the world, including Europe (Fuzzi et al. 2015). Such secondary aerosol is subdivided into secondary organic aerosol (SOA) and secondary inorganic aerosol (SIA), with different proportions depending on the different areas of the world and the source distribution. Ammonium sulphate and nitrate comprise practically the whole SIA mass and represent a major component of the fine PM mass. Contributions from the domestic sector (mainly household heating) are considered as the total of primary and secondary particles.

According to the TSAP Report, ammonia emissions, combined with traffic and industry emissions, are the main responsible for the secondary PM_2.5_ levels measured at European urban sites.

This evidence is connected to the ammonia emission whose main source is the agricultural sector (European Environment Agency 2018b). In 2016, the EU-28 agricultural sector emitted a total of 3.91 Mton of ammonia and was the source of 92% of total ammonia emissions across the region (European Environmental Agency 2018b). PM precursor nitrogen and sulphur oxides have been targeted at the EU scale relatively successfully since the 1990s, but ammonia has been neglected and not yet targeted stringently. Improving the practices of the agricultural and livestock sector and/or reducing the meat consumption could contribute significantly to EU air quality mitigation efforts (Bittman et al. 2014). In fact, the reduction of ammonia emissions is reported as the most effective way to reduce fine aerosol mass concentrations in Europe (Megaritis et al. 2013; Tschofen et al. 2019).

In spite of the improvements of air quality observed in Europe over the last 30 years, emissions from the agricultural sector have exhibited the lowest decrease, and the revised 2016 NECD has failed to efficiently tackle the problem, requiring EU member states to cut ammonia emissions by only 19% by 2030, relative to 2005 levels. This overall reduction effort is relatively low compared to the SO_2_ and NO_*X*_ prescribed cuts. Actually, over the period 2014–2017, an overall increase of European ammonia emissions of about 2.5% has been recorded, as a consequence of the lack of emission control in the agriculture sector (European Environment Agency 2019b).

The mismatch between the perceived and the actual sources of air pollution pointed out by our findings echoes the results of previous investigations. Research contributions on this topic focusing on other countries not included in this study show that people perceive traffic as the major source of air pollution, and that industry is also viewed as another significant source of pollution (Geelen et al. 2013; Liao et al. 2015; Cisneros et al. 2017).

## Science in the public debate on air pollution

The dissemination of scientific knowledge is an essential feature of the relationship between science and society. Research results have to be mainstreamed into the public debate, and to do so, the scientific message has to be conveyed in an accessible format and easy-to-understand language. Indeed, it has been emphasised that if public awareness about the impact of polluting agents on human health and air quality would improve, people and institutions are more likely willing to modify their attitudes (Kelly and Fussell 2015). Unfortunately, scientists are often reluctant to take a more active role in shaping the public understanding of science. Nevertheless, scientific dissemination targeting laypeople is crucial, since they possess only a limited understanding of the underlying scientific issues (Hendriks et al. 2016). In this respect, a Eurobarometer survey shows that European citizens believe that scientists should communicate more effectively (European Commission 2010).

Concerning air quality, research shows that laypeople have neither the right knowledge about the effects of air pollution and their health implications nor the appropriate information about air quality (Kelly and Fussell 2015). Scientific results must therefore be communicated in a clear, jargon-free way, to be fully accessible to non-experts (e.g. Gascoigne and Metcalfe 1997; Office of Science and Technology and the Welcome Trust 2001). Research also shows that people often lack the competence for differentiating misinformation from true information (Braten et al. 2011; Scheufele and Krause 2019). Indeed, as Kelly and Fussell (2015) point out, people’s understanding of air quality and its impact on health depends not only on the accessibility of the information, but also on the level of ‘understanding, perception and vested interest’ involved.

Since individuals are ‘cognitive misers’ possessing minimal knowledge allowing to take a stand on grand policy problems, they often employ ‘heuristic devices or mental shortcuts’, such as ‘group identification and social identity cues’ (Hart and Nisbet 2012). As a consequence, it has been argued that making scientific messages more accessible would not be enough to break the link between personal opinions and the human need of group identity (Kahan et al. 2012).

On the other hand, the exponential growth in the amount of available information (e.g. from web sources and social media) does not prevent misinterpretations. By way of contrast, the overload of contradictory and sometimes false information available online, coupled with the complexity and expertise required to understand the issues at stake, as in the case of air quality, has produced the so-called post-truth era (Keyes 2004). As Iyengar and Massey (2019) warn, ‘the ready availability of misleading and biased information in the media, often inserted deliberately by unscrupulous actors with ulterior motives’ may have caused growing scepticism about scientific evidence. Indeed, the degree of trust in science has decreased in the EU from 78% (in 2005) to 66% (in 2010) (European Commission 2010). In this respect, a Eurobarometer survey shows that 58% of the people believe that scientists can no longer be trusted on controversial scientific and technological issues because they depend more and more on money from industry, while only 16% disagree with this statement. In fact, some scientific topics, as is the case of most environmental issues, have become contentious, since they have been heavily politicised and pushed on the social media (Lewandowsky et al. 2013; Funk 2017).

Against this backdrop, translating complex scientific evidence into understandable facts for the public is crucial and communicating information about the scientifically based sources of air pollution can help raising awareness and changing citizens’ behaviours and attitudes (Zsoka et al. 2013; Pothithou et al. 2017).

## Conclusions

Our study shows that the majority of the population in the seven European countries we surveyed has little knowledge of the major sources of air pollutants. Our analysis of perceived polluting sectors has shown that people see industry and vehicular traffic as the most relevant sources of air pollutants. Our findings also indicate that respondents’ education, age, place of residence or gender influence only to a small extent the perception of air pollution causes. This suggests that the lack of information and knowledge about the causes of air pollution is widespread across different socio-economic groups and countries and that even respondents with higher levels of education are not fully aware of the actual sources of air pollution.

Among the mismatches between individual perception and real-world data, the role of emissions from the agricultural sector stands out, being constantly underestimated. This may, at least in part, be attributed to a stereotype of the countryside as a good place to live and/or as a repository of values (Shucksmith 2018), with the typical binaries in people’s mind being rural = peace/urban = noise, rural = slow/urban = fast, rural = clean/urban = dirty (Bell 2006). It may therefore not be surprising that a vast majority of the respondents in our survey did not identify the agri-food sector as an important contributor to air pollution.

Rectifying misperceptions could improve citizens’ views and attitudes, since individual perception and knowledge is a necessary (although not sufficient) component to any behavioural change (Fiedling and Head 2011; Levine and Strube 2012). To achieve this, there is a need for appropriate communication strategies addressing multiple targets. On one side, scientific information should be provided in the right format by the scientific community to both policymakers and the public. In particular, Iyengar and Massey (2019), reflecting upon the reception of scientific evidence in the post-truth society, suggest that, to curb public distrust in science, scientists should take an active role to prevent the spread of false scientific news through the use of digital arenas to immediately debunk fake facts. On the other hand, equally important is the role of the media in turning on a spotlight on crucial/impactful issues such as the environmental ones, and in producing the information in a way that people understand (Crow and Boykoff 2014).

## Footnotes

1 https://ec.europa.eu/eurostat/web/rural-development/methodology (Accessed 24/09/2020).

2 The category ‘others’ was not included in the data analysis.
